# From the lab to lifestyle: epigenetic clocks in personalized aging and health

**DOI:** 10.1007/s10522-026-10447-8

**Published:** 2026-05-06

**Authors:** José Santiago Ibáñez-Cabellos, José Luis García-Giménez, Javier Escobar, Federico V. Pallardó, Salvador Mena-Mollá

**Affiliations:** 1 Department of Physiology, Faculty of Pharmacy, University of Valencia, 46100 Burjassot, Spain; 2 EpiDisease S.L (Spin-Off From the CIBER-ISCIII), Parc Científic de La Universitat de Valencia, 46980 Paterna, Spain; 3 Department of Physiology, Faculty of Medicine and Dentistry, University of Valencia, 46010 Valencia, Spain; 4 Consortium Center for Biomedical Network Research On Rare Diseases (CIBERER), Institute of Health Carlos III, 46010 Valencia, Spain; 5 INCLIVA Health Research Institute, INCLIVA, 46010 Valencia, Spain; 6 OverGenes S.L, Parc Científic de La Universitat de Valencia, 46980 Paterna, Spain

**Keywords:** Aging, Epigenetics, DNA methylation, Epigenetic clocks, Biological age

## Abstract

Aging is a complex biological process characterized by progressive functional decline and increased risk of chronic diseases. In recent years, DNA methylation-based epigenetic clocks have emerged as some of the most robust biomarkers for estimating biological age. Initial research clocks, such as those developed by Horvath and Hannum, provided highly accurate chronological age predictions. Subsequent models, including PhenoAge, GrimAge, and DunedinPACE, improved upon this by incorporating health-related variables and functional measures, expanding their relevance to disease risk and pace of aging. Importantly, these multi-CpG clocks have demonstrated strong predictive accuracy, but their reliance on large numbers of CpG sites and high-throughput technologies limits their clinical scalability due to cost, complexity, and sample processing requirements. In this review, we critically evaluate the current landscape research-based epigenetic clocks, and their transition into direct-to-consumer testing. We discuss their key strengths, limitations, and translational potential, with particular emphasis on the growing demand for simplified, cost-effective, and analytically accessible epigenetic clocks, which should maintain predictive accuracy while enabling broader implementation in clinical and epidemiological settings. Special attention is given to *ELOVL2*-based clocks, which exemplify minimalistic yet robust models that can facilitate large-scale studies and democratize access to biological aging assessment. Ultimately, we argue that the next generation of epigenetic clocks should prioritize both analytical simplicity and validation across diverse populations to support personalized interventions for healthy aging.

## Chronological aging vs biological aging

Aging is characterized by a progressive decline in physiological functions, leading to increased vulnerability to diseases. This decline involves various interconnected molecular, cellular, and systemic changes that manifest as hallmarks of aging, including genomic instability, telomere attrition, epigenetic alterations, mitochondrial dysfunction, loss of proteostasis, cellular senescence, disabled macroautophagy, deregulated nutrient-sensing, stem cell exhaustion, altered intracellular communication, chronic inflammation and dysbiosis (López-Otín et al. 2023). These processes contribute to the deterioration of tissue function and an increased susceptibility to age-related diseases such as cardiovascular disorders, neurodegenerative conditions, cancer, immunosuppression and musculoskeletal diseases like osteoporosis and arthritis, among others. Understanding the mechanisms underlying these hallmarks provides insight into therapeutic strategies that may decelerate or reverse aspects of aging and its associated pathologies (López-Otín et al. 2023).

Aging itself is a major contributing factor to the prevalence of diseases in older adults. The concept of "biological age," which provides a more precise measure of an individual's aging process compared to "chronological age," was first introduced in 1947 by H. Benjamin (Benjamin 1947), and then developed by Baker and Sprott in 1988 (Baker and Sprott 1988). Unlike chronological age, which simply reflects the time elapsed since birth, biological age accounts for the physiological and functional state of an individual, offering a more accurate assessment of aging and its impact on health.

To understand and address the complexities of aging, it is crucial to focus on biological aging rather than merely cataloging age-associated alterations. In this regard, biological age can better reflect the diversity of the aging process across different individuals as it mirrors how a person is actually aging according to many different internal and external factors. The research on biological age does not replace the investigation of the underlying mechanisms that drive age-related diseases (Ikram 2024). Biological aging may help to provide a mechanistic understanding of aging manifestation. Determining biological aging, especially through standardized metrics or by using biological clocks, is essential for quantifying the functional decline in organisms and distinguishing it from chronological aging. Aging is not a direct cause of disease; rather, it is a summatory process of accumulated risk factors. In any case, the disparity between biological and chronological age offers critical insights into the effectiveness of interventions that can accelerate or decelerate aging, shedding light on the contributions of specific aging hallmarks. For example, physiological assessments (e.g., respirometry for energy expenditure), functional tests (e.g., sensory, psychomotor, and cognitive evaluations), and advanced "omics" technologies (e.g., genomics, epigenomics, and metabolomics), often at the single-cell level, are contributing to evaluate biological aging and provide further insights in the events associated to aging. These approaches enable the evaluation of health deterioration patterns, to better predict age-related conditions, including mortality, and the efficacy of anti-aging strategies, paving the way for targeted interventions to promote healthy aging (López-Otín et al. 2023).

In recent years, significant progress has been made in estimating biological age through a variety of biomarkers, including telomere length, DNA methylation, proteomics, metabolomics, glycomics, wearable sensor data, and blood-based clinical biomarkers (Pyrkov et al. 2021; Jylhävä et al. 2017; Horvath and Raj 2018; Frenck et al. 1998; Macdonald-Dunlop et al. 2022; Oh et al. 2023; Krištić et al. 2014). One of the first molecular tools developed to assess a biological clock was the measurement of the length of telomeres, the repetitive DNA structures at the ends of chromosomes, which shorten with each cell division cycle. This shortening mechanism is associated with cellular ageing and declining health (Levy et al. 1992). Different methods have been developed to measure telomere length. However, current methods to measure telomere length have some variability that could affect results (Lai et al. 2018; Yu et al. 2024). On the other hand, there are also epidemiological studies showing contradictory results between telomere shortening and age (Vaiserman and Krasnienkov 2021). Protein-based aging clocks have demonstrated high accuracy in predicting chronological age (correlations r = 0.92–0.94) and have been associated with the incidence of several chronic diseases (Argentieri et al. 2024). Zhang et al. (Zhang et al. 2024) developed a plasma metabolomic aging score with a high predictive power for short-term (1–5 year) mortality. Similarly, an independent study (Chen et al. 2024) identified several of these biomarkers as part of a distinct score positively associated with biological aging, highlighting key areas of overlap—particularly in functional categories such as systemic inflammation (glycoprotein acetyls-GlycA), energy metabolism (glucose, lactate, glycemia), lipid profile (polyunsaturated and saturated fatty acids), and amino acid-related pathways (including kynurenine and general amino acid metabolism). This convergence across independent models underscores the central role of these metabolic processes in aging biology. Wearable sensors for biological age estimation typically measure physical activity, physiological signals, and even biochemical markers (like as sweat) in real-time leading to a biological age score that could be associated with mortality risk, healthspan, and the efficacy of interventions such as nutrition or medication (McIntyre et al. 2021; Niederberger et al. 2023). Even, composite blood-based biomarkers have demonstrated the ability to detect differences in biological age, even in young and healthy individuals, before the onset of disease or visible signs of accelerated aging (Belsky et al. 2015). Other biological clocks have combined multiple biomarkers including clinical variables obtained from blood analysis, hematology, anthropometry, organ function test measurements, functional aging index or frailty index (Li et al. 2023; Vaiserman and Krasnienkov 2021).

Despite these advancements, DNA methylation-based biomarkers continue to dominate aging research due to their superior precision, broader applicability across tissues and species, and stronger correlations with health and disease. These advantages make them especially useful for longitudinal and comparative studies in both humans and animal models.

Within this framework, epigenetic changes play a fundamental role in cellular aging by mediating the interaction between environmental factors and gene expression, thereby influencing the organism's response to environmental stressors (Fraga and Esteller 2007; Benayoun et al. 2015).

## Epigenetic alterations as one of the main hallmarks of aging

Epigenetic changes are recognized as key contributors to the aging process, influencing gene expression patterns and cellular function without altering the underlying DNA sequence (López-Otín et al. 2013, 2023). The most studied alterations, four stand out for their impact on aging and their utility in assessing biological age: DNA methylation dysregulation, post-translational modifications (PTMs) of histones, microRNA (miRNA) signatures, and chromatin structure remodeling. Among these epigenetic mechanisms, DNA methylation has emerged as a critical element in the aging process, serving as both a marker and a regulator of age-related biological functions (Bell et al. 2019; Fraga et al. 2005; Hannum et al. 2013; Horvath 2013).

DNA methylation patterns undergo profound changes with age, characterized by global hypomethylation and site-specific hypermethylation, particularly at CpG islands (Ciccarone et al. 2018). Based on this evidence, the concept of the 'epigenetic clock' was introduced in 2013 by Steve Horvath to associate DNA methylation with biological age, defining the most well-known epigenetic clock, the Horvath Clock (Horvath 2013). At the same time, it is not the only clock built using an approach based on epigenetic markers, and other epigenetic clocks focused on mechanisms distinct to DNA methylation, such as those based on histone posttranslational modifications (PTMs) have been also described.

Particularly, acetylation, methylation, and phosphorylation, are critical in aging as they regulate chromatin accessibility and transcriptional activity (Yi and Kim 2020; Wang et al. 2018). Interestingly, aging is linked to a decline in histone acetylation, contributing to reduced gene expression and cellular senescence (Ryan and Cristofalo 1972). Most characterized deacetylase mechanisms are those mediated by Sirtuins (SIRT1–7), considered Class III NAD⁺-histone deacetylases (HDACs), which are involved in key processes related to aging, including metabolism and DNA repair (Longo and Kennedy 2006). Therefore, strategies aimed at activating these enzymes have been investigated as potential anti-aging interventions. Among these strategies, various molecules such as resveratrol have been proposed (Grabowska et al. 2017); in contrast, calorie restriction is the only intervention that has consistently demonstrated efficacy to date(Kittana et al. 2024). Although sirtuin genes are closely associated with longevity and cellular regulation, they cannot currently be used to determine the individual's biological age (Mathur et al. 2024).

Benayoun et al. (Benayoun et al. 2019) studied in mice models the levels of histone H3 trimethylation at lysine 4 (H3K4me3), a promoter-associated mark, and histone H3 acetylation at lysine 27 (H3K27ac), an enhancer-associated mark. The analysis of these marks revealed distinct epigenomic and transcriptomic landscapes differentiating between young and aged samples. Machine learning approaches further demonstrated that specific epigenomic states can predict transcriptional changes associated with aging. These findings highlight how histone modifications not only impact chromatin structure but also mediate inflammatory responses and other transcriptional shifts observed with aging.

miRNAs, small non-coding RNAs, modulate gene expression post-transcriptionally and exhibit age-dependent changes in their expression profiles. Huan et al. (Huan et al. 2018) identified 127 miRNAs with differential age-associated expression in a peripheral blood analysis of more than 5,000 adults. This study showed that most of these miRNAs are underexpressed in older individuals, suggesting their influence on the regulation of transcriptional programs and RNA processing. In addition, they developed a model for predicting biological age based on miRNAs (‘microRNA age’) that correlates modestly with epigenetic clocks based on DNA methylation and mRNA expression and was also associated with age-related diseases such as hypertension and coronary heart disease. Another study by Roig-Genoves et al. (Roig-Genoves et al. 2024) designed an epigenetic clock based on miRNA expression profiles to predict the biological age of the skin, one of the visible signs of the most exposed tissues to human ageing. Their model was able to classify skin samples into age ranges with an accuracy of 80% and predicted age with a mean absolute error of 10.89 years, which is close to other biological clocks based on transcriptomics. These advances underline the potential of miRNA signatures as useful tools both for predicting accelerated ageing and for application in the pharmaceutical and dermocosmetics industry.

Finally, changes in chromatin architecture, including the loss of heterochromatin and increased chromatin relaxation, also impair nuclear organization and gene expression fidelity during aging. Rechsteiner et al. (Rechsteiner et al. 2022) developed a novel aging clock based on chromatin accessibility using profiles derived from peripheral blood mononuclear cells of 157 human donors. This clock demonstrated remarkable accuracy, with a median absolute error of 5.69 years. By comparing chromatin accessibility data with matched transcriptomic profiles, the study showed that genomic sites used for age prediction undergo transcriptional changes during aging. This chromatin accessibility clock bridges the gap between epigenetic alterations and their downstream biological effects, providing a powerful tool to investigate how aged epigenetic states affect cellular function.

These epigenetic alterations collectively reflect the complex interplay of molecular mechanisms driving aging, offering robust frameworks for evaluating biological age. Thus, they can be use as biomarkers to predict morbidity and mortality (Duan et al. 2022; Levine 2020; Beynon et al. 2022). Future research should aim to integrate these markers into comprehensive models that improve aging predictions and therapeutic interventions.

## DNA methylation-based clocks

Two main generations of methylation clocks can be distinguished, which represent progressive improvements in model design, predictive capacity, and clinical relevance for aging and disease outcomes.

First Generation clocks were developed to estimate chronological age based solely on DNA methylation patterns at selected CpG sites. They are highly accurate at predicting actual age but less effective at capturing biological aging or health-related outcomes beyond age itself (Crimmins et al. 2021; Sugden et al. 2022; Tay et al. 2025; Di Lena et al. 2022; Bell et al. 2019; Noroozi et al. 2021; Field et al. 2018; Zheng et al. 2024).

The Horvath Clock, developed in 2013, is a widely recognized and precise tool for estimating epigenetic age across multiple tissue types, utilizing 353CpG sites distributed across the genome (Durso et al. 2017; Horvath 2013) with a correlation of 0.96 between epigenetic and chronological age, and is applicable to a variety of tissues, although limited in cultured cells (Horvath 2013). Validated in studies on human aging, it has proven to be effective in detecting epigenetic age acceleration in conditions such as Down syndrome and neurodegenerative diseases (Hannum et al. 2013). The model’s versatility lies in its multi-tissue applicability, making it ideal for large-scale studies on diverse populations, with applications in tissues such as blood, skin, and brain. Recent studies, using the DNAmFitAge variant, have linked it to physical fitness and quality of life (Kerepesi and Gladyshev 2023). This clock shows limitations in accuracy when applied to certain tissues, diverse populations and age extremes, as well as in the ability to reflect actual biological ageing. In addition, its usefulness may be affected by the cellular composition of the sample and updates in methylation technology platforms (Table 1).

**Table 1:** Comparative table of the main published DNA methylation-based epigenetic clocks. The table includes the Clock name, the Generation classification (first or second), the year of publication, the model’s performance in terms of the Pearson correlation coefficient (R) between predicted and chronological age, the mean absolute error in years (MAE), the Age Range of the population used for model training and validation, the number of samples used for model development (N Training) and for independent validation (N Validation), the total number of CpG sites (Nº CpGs) included in the final model, the primary Predicted Outcome, and the main pathophysiological or clinical conditions associated with each clock across different studies

Clock	Generation	Year	R	MAE	Age range	N training	N validation	Nº CpGs	Predicted outcome	Associated conditions
Horvath Clock (Horvath 2013)	First	2013	0.96ᵃ	3.6ᵃ	0–100	3,931ᵇ	3,211	353	Biological Age	Down syndrome, neurodegenerative diseases, cancer, cardiovascular diseases
Hannum Clock (Hannum et al. 2013)	First	2013	0.91ᶜ	4.9ᶜ	19–101	482	174	71	Biological Age	Cardiovascular risk factors, cancer
DNAm PhenoAge (Levine et al. 2018)	Second	2018	0.94ᵈ	N/Rᵉ	21–100	456	10,758ᶠ	513	Phenotypic Age	All-cause mortality, mortality from age-related diseases, cardiovascular disease (CVD) and coronary heart disease (CHD) mortality, cancer occurrence and mortality, as well as Alzheimer’s disease
GrimAge (Ake T. Lu et al. 2019)	Second	2019	0.82ᵍ	N/Rʰ	40–92	1,731	6,935ⁱ	1,030	Mortality Risk	Precise estimation of time-to-death, time-to-cancer, time-to-cardiovascular diseases (CVD), time-to-fatty liver, and time-to-menopause
DunedinPACE (Belsky et al. 2022)	Second	2022	0.78ʲ	N/R	26–45ᵏ	1,037	6,111ˡ	173	Pace of Biological Aging	Morbidity, mortality, disability, cognitive decline
CheekAge (Shokhirev et al. 2024)	Second	2024	0.95ᵐ	3.22	18–93	8,045ⁿ	225°	10,000 clusters (~ 200,000 filtered CpGs)	Biological Age	Obesity (BMI), alcohol consumption, smoking, physical activity, sleep quality, and mental health factors

ᵃ Performance in independent test data (datasets 40–71). Training performance: r = 0.97, MAE = 2.9 years (Horvath 2013)

ᵇ From a total of 7,844 non-cancer samples across 82 datasets: 3,931 for training (datasets 1–39) and 3,211 for testing (datasets 40–71). Exact training N confirmed from Additional File 1 (Horvath 2013)

ᶜ Performance in the validation cohort (N = 174). Training performance: r = 0.96, MAE = 3.9 years ((Hannum et al. 2013)

ᵈ r = 0.94 represents the correlation between phenotypic age (derived from clinical biomarkers in NHANES III) and chronological age. DNAm PhenoAge correlations with chronological age vary across validation cohorts (Levine et al. 2018)

ᵉ MAE for DNAm PhenoAge is not explicitly reported in the main text of the original publication. N/R = Not Reported (Levine et al. 2018)

ᶠ Multiple external validation cohorts: WHI emulation (n = 1,140), WHI AS265 (n = 2,107), NHANES IV (n ≈ 2,500), FHS (n = 2,553), NAS (n = 657), JHS (n = 1,801). Total validation N = 10,758 (Levine et al. 2018)

ᵍ r = 0.82 in the FHS test set. Across all validation cohorts r ≥ 0.79 (Ake T. Lu et al. 2019)

ʰ GrimAge was not evaluated using MAE in the original publication; predictive performance was assessed using Cox regression hazard ratios for mortality and disease outcomes. N/R = Not Reported (Ake T. Lu et al. 2019)

ⁱ Total validation including FHS internal test (n = 625) and external cohorts: WHI BA23, WHI EMPC, JHS, and InCHIANTI. Total validation N = 6,935 individuals (7,375 arrays) (Ake T. Lu et al. 2019)

ʲ In-sample correlation between DunedinPACE and the 20-year longitudinal Pace of Aging measure, not a prediction of chronological age (Belsky et al. 2022)

ᵏ The Dunedin Study is a single-year birth cohort (1972–73). The age range 26–45 reflects the four assessment ages at which the 19 biomarkers were longitudinally measured. DunedinPACE was externally validated in cohorts spanning ages 18–95 (Belsky et al. 2022)

ˡ External validation cohorts: GSE55763 (n = 36, test–retest replicates), Understanding Society (n = 1,175), NAS (n = 771), FHS Offspring (n = 2,471), and E-Risk (n = 1,658). Total = 6,111 (Belsky et al. 2022)

ᵐ Reported as R^2^ = 0.91 in the original publication (tenfold cross-validation); r = √0.91 ≈ 0.95 (Shokhirev et al. 2024)

ⁿ The 8,045 samples were used for model development using tenfold cross-validation with a simulated annealing optimization approach repeated 1,098 times; no fixed external training/test split was employed (Shokhirev et al. 2024)

° External validation in an independent buccal dataset (N = 225) and 10 publicly available MethylationEPIC datasets from GEO including blood, skin, fibroblasts, rectal, and epithelial tissue samples (Shokhirev et al. 2024)

The Hannum Clock focuses specifically on DNA methylation in blood, analyzing approximately 71 CpG sites to estimate biological age (Hannum et al. 2013). Although this epigenetic clock was originally designed to measure chonological age, it has proven particularly effective in studies involving older adults, demonstrating a strong correlations with mortality associated-risk factors (Dhingra et al. 2018; Armstrong et al. 2017). It use, however, is limited to blood samples, reducing its utility in multi-tissue studies, and its predictive sensitivity is less robust in younger populations or diverse health conditions (Table 1).

Second‑generation, or next‑generation, epigenetic clocks extend first‑generation age estimators by incorporating health‑related variables (for example, clinical blood biomarkers and lifestyle factors such as smoking) into the training framework alongside DNA methylation data, thereby improving the prediction of mortality, disease risk, and healthspan compared with first‑generation clocks (Crimmins et al. 2021; Sugden et al. 2022; Tay et al. 2025; Di Lena et al. 2022; Bell et al. 2019; Noroozi et al. 2021; Field et al. 2018; Zheng et al. 2024). These models show stronger associations with physical function, frailty, disability, cognitive performance, incident age‑related diseases and dementia (Crimmins et al. 2021; Sugden et al. 2022; Tay et al. 2025; Di Lena et al. 2022; Bell et al. 2019; Noroozi et al. 2021; Field et al. 2018; Zheng et al. 2024; Ikram 2024; Belsky et al. 2022). Representative examples include DNAm PhenoAge and GrimAge, which are trained on composite morbidity and mortality outcomes and show robust links with all‑cause mortality and cardiovascular and neurodegenerative diseases (Levine et al. 2018; Ake T. Lu et al. 2019; McCrory et al. 2021). More recent clocks such as DunedinPACE quantify the pace of biological aging from longitudinal multisystem biomarkers (Belsky et al. 2022), whereas CheekAge represents a buccal‑based next‑generation clock designed to capture health and lifestyle‑related variation in biological age (Shokhirev et al. 2024; Wu et al. 2025).

The DNAm PhenoAge clock, developed by Levine et al., integrates DNA methylation data with clinical biomarkers to estimate a “phenotypic age” reflecting biological age and overall physiological health (Levine et al. 2018). This clock utilizes 513 CpG sites selected through an Elastic Net regression model to predict phenotypic age, which incorporates metrics like inflammation, immune function, and metabolic health from NHANES III data. DNAm PhenoAge has shown strong associations with all-cause mortality, aging-related diseases (e.g., cardiovascular disease, diabetes, chronic respiratory diseases), and physical and cognitive functioning. It surpasses first generation epigenetic clocks, such as Horvath’s and Hannum’s, in predicting morbidity and mortality by capturing deviations in biological aging among same-aged individuals. Advantages of the DNAm PhenoAge include its applicability across tissues, its robustness in identifying aging-related risks, and its ability to differentiate individuals based on healthspan (Table 1). In the original publication, DNAm PhenoAge was primarily evaluated through its associations with morbidity and mortality, and the authors did not report a mean absolute error for the prediction of chronological age, which is why no mean absolute error (MAE) value is provided for this clock in Table 1 (Levine et al. 2018).

GrimAge incorporates additional biomarkers, such as plasma proteins and smoking history, to predict long-term health risks, mortality, and life expectancy (Ake T. Lu et al. 2019). It combines epigenetic markers with health risk factors, achieving high precision in predicting mortality and disease risk, particularly in populations with significant health risks. GrimAge has been validated in large cohorts and can be used in preventive medicine to identify individuals at high risk for age-related diseases, including cardiovascular conditions. Even more, the GrimAge represents a step-improvement in the use of the epigenetic clocks for identifying age-related decline compared to Horvath, Hannum, PhenoAge clocks (McCrory et al. 2021).

Despite these advantages, its implementation requires detailed lifestyle data and advanced platforms for protein and methylation analysis (Table 1). Importantly, the original GrimAge article focused on Cox proportional hazards models for mortality and disease outcomes and did not report a mean absolute error for age prediction; therefore, an MAE value is not included for this clock in Table 1 (Ake T. Lu et al. 2019).

The DunedinPACE Clock, developed by Belsky et al. (Belsky et al. 2022), is a DNA methylation-based model specifically designed to measure the pace of biological aging. It builds upon the earlier DunedinPoAm model and quantifies longitudinal changes in multi-system organ integrity using 19 biomarkers, including cardiovascular, metabolic, renal, and immune systems, collected over two decades from the Dunedin cohort (Belsky et al. 2022). The algorithm uses Elastic Net regression on 173 CpG sites, selected for their high test–retest reliability, to estimate the pace of aging from a single blood sample. DunedinPACE clock demonstrated strong correlations with morbidity, disability, and mortality, showing predictive value for functional decline, cognitive aging, and early-life adversities. Compared to other clocks, like GrimAge or Horvath’s clock, DunedinPACE uniquely captures the rate of aging rather than cumulative age-related changes. Its high precision, reliability due to an intraclass correlation coefficient (ICC) of 0.96, and robustness to smoking-related bias make it a valuable tool for trials and longitudinal studies exploring interventions that target the fundamental mechanisms of aging to prevent or delay the onset of age-related diseases and decline. This development was based on a New Zealand cohort, and although it has been tested in thousands of people from Europe (Janssens et al. 2025; Mak et al. 2024), the United States (Bozack et al. 2025); (Whitman et al. 2024) and China (Miao et al. 2024), most studies have been conducted in populations of European and Asian ancestry, so additional validation in more diverse populations is needed. Furthermore, its implementation requires high-density methylation arrays, which may limit its accessibility in some settings (Belsky et al. 2022) (Table 1). DunedinPACE was developed to quantify the rate of biological aging rather than chronological age itself; accordingly, the original publication reports correlations with the longitudinal Pace of Aging and test–retest reliability, but no mean absolute error in years, so the MAE entry is marked as not applicable in Table 1 (Belsky et al. 2022).

The CheekAge Clock, developed as a next-generation epigenetic aging clock (Wu et al. 2025), is specifically designed to estimate biological age using DNA methylation data from buccal epithelial cells (Shokhirev et al. 2024). This clock was trained on a dataset of over 8,000 individuals aged 18–93 years and leverages 10,000 CpG clusters derived from approximately 200,000 filtered CpGs to predict biological age and detect age acceleration linked to lifestyle and health factors such as diet, physical activity, sleep, and mental well-being. The model employs a novel simulated annealing approach, a stochastic global optimization strategy that iteratively explores the solution space to find optimal linear models. This approach was combined with CpG filtering, CpG clustering, and clock ensembling. Specifically, the simulated annealing process optimized a custom objective function balancing model accuracy, the significance of correlations between delta age and lifestyle/health survey factors, and model complexity. The training was repeated 1,098 times, and a weighted mean of the top 100 scoring models was used to produce the final CheekAge predictor. This ensemble methodology aims to reduce model‑specific noise and improve the reproducibility of biological age estimates derived from the methylation data. With a reported R^2^ of 0.91, a root mean squared error (RMSE) of 4.5 years, and a MAE of 3.22 years, CheekAge showed a strong predictive ability to estimate biological age and acceleration of ageing, as well as a significant association with mortality risk, but its ability to predict specific comorbidities is limited and its correlations with lifestyle factors are relatively weak (Shokhirev et al. 2024) (Table 1).

## From bench to bedside: clinical and population-uses of epigenetic clocks

There is growing interest in the potential clinical and wellness applications of epigenetic clocks. These clocks not only allow accurate estimation of biological age but also offer a unique opportunity to identify individual differences in the ageing process, which could play a crucial role in personalizing medical treatments and importantly, to provide lifestyle habits and actionable information which may improve the health status of patients. Their ability to correlate biological age with factors such as the risk of chronic diseases, the efficacy of specific therapies and the progression of certain pathologies positions epigenetic clocks as a transformative tool in precision medicine.

Reflecting this growing interest and clinical potential, several direct-to-consumer tests based on DNA methylation epigenetic clocks have emerged, such as NOVOS, TruAge, myDNAge and others (Table 2). These tests not only measure biological age and the rate of ageing, but in some cases integrate additional parameters, such as analysis of specific body systems and personalized recommendations for improving health and ageing. The main tests on the market are described below.

**Table 2:** Summary of epigenetic age prediction models currently available for public use. The table includes the name of the test (Test Name) and the underlying epigenetic Model it is based on. It specifies the biological Sample used, the number of CpG sites analyzed (Nº CpGs), the mean absolute error in years (MAE) and the coefficient of determination (R^2^) when available. Additional information includes the advantages and disadvantages associated with each test

Test name	Clock	Sample	R^2^	MAE	Nº CpGs	Advantages	Disadvantages
Novos age	DunedinPACE Clock	Blood	N/R^a^	N/R^a^	173	Uses DunedinPACE pace-of-aging biomarker; strong scientific backing (ICC > 0.96); provides ageing-rate and organ-system information	Invasive sampling; requires handling and shipping of blood; does not provide age in years but rather the rate of ageing; depends on methylation arrays, increasing cost and complexity
Index age	APEX	Saliva	N/R^b^	N/R^b^	> 100,000	Measures ageing in 9 systems; high precision in internal replication; saliva-based sampling	No peer-reviewed publication explicitly detailing the APEX algorithm and its validation; processing time and cost may limit accessibility
myDNAge	Horvath Clock + SWARM™	Blood/Urine	0.96^c^	3.6^c^	~ 2,000	Versatile (blood/urine); based on Horvath’s epigenetic clock; peer-reviewed evidence supporting the underlying DNAge™ clock	Invasive sampling for blood; requires methylation array technology; detailed performance metrics for the commercial implementation are not fully disclosed
DeepMAge	DeepMAge Clock	Blood	0.93^d^	2.77^d^	~ 1,000	Deep-learning-based model; good accuracy; detects age-related conditions and shows sensitivity to disease status	Requires invasive blood sampling; high computational demands; further validation is needed in diverse and longitudinal cohorts
TallyAge	CheekAge Clock	Buccal Swab	0.91^e^	3.87^e^	193	Non-invasive buccal sampling; based on the CheekAge epigenetic clock; supported by peer-reviewed validation; provides lifestyle-related ageing insights	Requires further longitudinal research; correlations with lifestyle factors are modest and may not fully explain variation in epigenetic age
TruAge	DunedinPACE Clock	Blood	N/R^a^	N/R^a^	173	Uses DunedinPACE to estimate ageing rate and multiple organ-system scores; strong scientific backing (ICC > 0.96); includes additional biomarkers such as telomere length and immune cell ratios; academically validated model	Invasive capillary blood sampling; requires handling and shipping of blood; does not provide age in years but rather the rate of ageing; relatively high cost
DoNotAge	DoNotAge Clock	Saliva	N/R^f^	N/R^f^	N/R	Non-invasive saliva-based sampling; provides biological age, inflammation metrics and system-specific scores	Underlying clock algorithm and validation are not disclosed in peer-reviewed publications; limited transparency about CpG selection and performance metrics
EpiAge	EpiAge Clock	Saliva	N/R^f^	N/R^f^	300	Simple, non-invasive saliva collection; targeted CpG panel	No independent peer-reviewed validation of the commercial test; correlation and MAE data are not publicly available
Chronomics	Chronomics Clock	Saliva	N/R^f^	N/R^f^	N/R	Affordable, non-invasive test focusing on lifestyle and environmental factors; user-friendly online dashboard	No peer-reviewed description of the specific epigenetic algorithm; limited transparency regarding CpG sites and modelling approach; lack of published accuracy metrics may hinder interpretation for individual users

^a^ Novos Age and TruAge (DunedinPACE): N/R as it measures pace of aging (biological aging rate per chronological year), not chronological age directly. ICC ≈ 0.96 refers to test–retest reliability (Belsky et al. 2022)

^b^ Index Age (APEX): Metrics not reported in peer-reviewed publications specific to the commercial test. (Higgins-Chen et al. 2022) demonstrates improved reliability for standard clocks (~ 1.5 years replicate agreement), but APEX algorithm details remain proprietary

^c^ myDNAge (Horvath + SWARM™): MAE = 3.6 years, r ≈ 0.96 (R^2^ ≈ 0.92) from Horvath's original pan-tissue clock (2013); commercial implementation lacks independent validation metrics (Horvath 2013)

^d^ DeepMAge: Median absolute error (MedAE) = 2.77 years (independent verification cohort, n = 1,293); r = 0.97 (R^2^ ≈ 0.93); 1,000 CpGs (Galkin et al. 2021)

^e^ TallyAge (CheekAge): MAE = 3.87 years, R^2^ = 0.91 for 450 K array version; full EPIC array: MAE = 3.05 years, R^2^ = 0.93 (Shokhirev et al. 2024)

^f^ Chronomics, DoNotAge, EpiAge: N/R; no peer-reviewed publications detailing commercial algorithms, CpG selection, or performance metrics. Note: EpiAgePublic (academic model, 3 CpGs ELOVL2, r = 0.87) is distinct from commercial EpiAge

The **Novos Age Clock** (www.novoslabs.com), developed by NOVOS Labs, estimates biological age and the pace of ageing (biological aging rate) by applying the DunedinPACE epigenetic biomarker to DNA methylation data from a capillary blood sample collected via finger‑prick. DunedinPACE was trained in the Dunedin Study as a measure of the rate of biological ageing over 20 years and uses 173 CpG sites selected for high test–retest reliability, showing excellent technical reproducibility (intraclass correlation coefficient ≈ 0.96) and robust associations with morbidity, disability and mortality. The test report links these estimates to lifestyle factors such as diet, exercise and stress management and emphasizes actionable recommendations. The use of a well‑validated pace‑of‑ageing algorithm is a strength, but reliance on array‑based DNA methylation profiling entails higher experimental and computational costs and contributes to relatively high pricing and processing times, and its current availability is restricted to the US and Canada (Table 2).

The **Index Biological Age Test** (www.elysiumhealth.com), developed by Elysium Health in collaboration with Dr. Morgan Levine, is a saliva-based epigenetic clock that uses the proprietary Algorithmic Platform for Epigenetic Examination (APEX) and a custom Illumina‑based chip to analyse DNA methylation at over 100,000 CpG sites. This technology enables accurate measurement of biological age, the cumulative ageing rate and specific biological ages for nine systems, including the brain, heart, immune system and metabolic systems. The APEX algorithm has been peer‑reviewed (Higgins-Chen et al. 2022) and shown to markedly improve technical reliability of epigenetic age measures, with most replicates agreeing within 1.5 years and improved performance relative to first‑generation clocks. Even so,the reliance on array‑based methylation profiling, the six‑week processing time and the relatively high price may limit accessibility for some consumers (Table 2).

The **myDNAge® test** (https://www.mydnage.com), developed by Epimorphy, is based on Dr Horvath's epigenetic clock (Horvath 2013), a proprietary SWARM™ next‑generation sequencing workflow that quantifies DNA methylation at more than 2,000 genomic loci. The assay can be performed on blood or urine and reports an estimated biological age (DNAge) together with indices that contextualise age acceleration relative to peers and, in some reports, genetic information such as APOE and MTHFR genotypes. In Horvath’s original publication, the underlying DNAm age algorithm achieved a correlation of approximately 0.96 with chronological age and a mean absolute error of about 3.6 years across multiple tissues, and this clock has been extensively used in epidemiological studies linking epigenetic age acceleration to morbidity and mortality. Detailed performance metrics specific to the myDNAge commercial implementation have not been fully disclosed, and the test cost and processing time of 4—6 weeks represent practical limitations (Table 2).

The **DeepMAge Clock** (www.deeplongevity.com), developed by Deep Longevity,

is a DNA methylation‑based ageing clock trained with deep neural networks on array‑based blood methylation data. In the original publication, DeepMAge was trained on 4,930 blood DNA methylation profiles and achieved a cross‑validated median absolute error of 2.24 years in training datasets and 2.77 years in an independent healthy verification cohort of 1,293 samples, outperforming the 2013 Horvath clock in most validation studies. DeepMAge shows sensitivity to several age‑related conditions, including ovarian cancer, inflammatory bowel disease and multiple sclerosis, making it a potentially informative marker of health‑related ageing. Its complexity and the need for array‑based DNA methylation assays increase computational and experimental demands, and further longitudinal validation in diverse populations is required (Table 2).

The **Chronomics Epigenetic Biological Age Test** (www.chronomics.com), calculates biological age by methylating DNA from saliva samples, focusing on how lifestyle and environmental factors influence aging. It offers a clinically sound view of aging and associated health risks. The test emphasises non‑invasive, self‑administered sampling and presents results through an online dashboard that explains biomarker readouts and suggests lifestyle modifications. However, no dedicated peer‑reviewed publication currently details the specific algorithm, CpG sites, or accuracy metrics for the commercial Chronomics test, and the relatively long processing time of approximately 8—12 weeks furtherlimits independent evaluation of its performance and may be considered a practical drawback (Table 2).

The **TallyAge™ Test** (www.tallyhealth.com) is a commercial implementation of the CheekAge buccal epigenetic clock, designed to estimate biological age from DNA methylation in cheek‑cell samples. CheekAge was developed using MethylationEPIC data from more than 8,000 adults aged 18—93, and the final model comprises 193 CpG sites selected through a simulated annealing approach combined with CpG clustering and model ensembling. In internal validation, the clock achieved an R^2^ of 0.91 for chronological age, with a mean absolute error of 3.87 years (450 K array version), and age‑acceleration measures derived from CheekAge have been associated with a broad range of lifestyle and health variables as well as mortality risk in longitudinal cohorts. The buccal swab sample makes the assay non‑invasive and user‑friendly, and published analyses show that CheekAge acceleration correlates with a range of health factors and diseases (Shokhirev et al. 2024), although the effect sizes for individual lifestyle variables are modest and do not fully explain inter‑individual variation in epigenetic age. Further longitudinal studies are needed to characterise how this clock responds to interventions and to assess its utility for long‑term health monitoring (Table 2).

The **TruAge Complete Collection Clock** (www.trudiagnostic.com), developed by TruDiagnostic, is a commercial test that uses the DunedinPACE DNA methylation model to assess the rate of ageing and multiple system‑specific scores (Belsky et al. 2022). This test analyses DNA methylation at 173 CpG sites to estimate biological age, ageing rate and the biological age of 11 key organ systems, including the brain, heart and liver. The assay uses capillary blood obtained via a finger‑prick sample as biospecimen, reporting biological age, ageing rate and additional biomarkers such as telomere length and immune cell ratios. Published validations of DunedinPACE indicate strong correlations with longitudinal measures of biological ageing and health outcomes rather than with chronological age itself (Belsky et al. 2022), and the TruAge report applies these estimates to provide lifestyle‑oriented feedback. As with other finger‑prick-based assays, the sampling procedure is minimally invasive but may still be uncomfortable for some users and entails the usual constraints for handling and shipping blood samples, and the relatively high cost may limit accessibility for broad population use (Table 2).

The **DoNotAge Biological Age Test** (www.donotage.org) is an epigenetic clock, which like the previous tests, uses DNA methylation levels. The test uses non-invasive saliva samples to assess several CpG sites associated with ageing and health outcomes. Although specific details about the underlying clock model have not been publicly disclosed, the test provides comprehensive information, including biological age, inflammation scores and system-specific metrics such as Memory Age and Eye Age. The results are presented in a report that includes data related to health factors such as immunity, gut health, mental health and vitamin deficiencies. It is notable for its non-invasive procedure, and its holistic report with various health metrics. Its price is somewhat higher than other clocks. Analysis times are relatively long, often requiring several weeks, andthe absence of peer-reviewed publications validating the specific test model represents an additional limitation (Table 2).

The **EpiAge Test** (www.epi-age.de), from HKG EpiTherapeutics Laboratory, is a DNA methylation-based clock using saliva samples. The test measures methylation levels at 300 CpG sitesusing Illumina triple sequencing to provide an accurate assessment of biological age, reflecting the cumulative impact of genetic and environmental factors on the aging process. Advantages of the test include its simplicity, as saliva collection is non-invasive and easy to process, as well as iits ability to estimate biological age through targeted CpG analysis. Its relatively low cost further enhances accessibility for a wide range of users. Limitations include the lack of independent validation in peer-reviewed studies and the absence of reported correlation data or MAE values to assess the accuracy of the clock (Table 2).

As outlined above, the increasing number of commercial epigenetic clocks reflects a growing interest in this field, underscoring their dual role in both clinical and consumer applications. These clocks aim to move beyond mere chronological age estimation, offering insights into biological aging, aging pace, and related health risks. By utilizing DNA methylation data, they enable individuals to implement targeted lifestyle or therapeutic interventions to promote healthier aging trajectories. Despite their promise, several limitations remain. Many earlier-generation clocks require blood samples obtained via finger pricks, which can be uncomfortable and may reduce user compliance. Additionally, the reliance on methylation arrays often entails high computational demands, elevated costs, and long turnaround times, all of which can hinder widespread adoption.

To truly transform clinical and personal health monitoring, future epigenetic clocks should prioritize ease of use, minimal invasiveness, fast processing, and affordability, without compromising accuracy. Encouragingly, models like DunedinPACE show strong associations with aging biomarkers, and direct-to-consumer solutions based on this epigenetic clock may provide more detailed information on ageing dynamics than earlier implementations. Moreover, commercial tests such as EpiAge and TallyAge™ emphasize non-invasive sampling and user accessibility as a strength of the test (Table 2). Nonetheless, continued methodological improvements, cost reductions, and robust validation studies will be essential to unlock their full potential in advancing personalized aging management worldwide.

## Simplifying epigenetic aging: reduced-CpG models and the contribution of ELOVL2

Despite substantial progress in the development of highly accurate epigenetic clocks, most current models rely on the quantification of hundreds or thousands of CpG sites with high throughput platforms. This requirement entails complex methodologies, elevated costs, and technological infrastructure that may not be readily available outside specialized academic or clinical settings (Belsky et al. 2018). Such limitations constrain their applicability in large-scale epidemiological studies, low-resource environments, and preventive healthcare contexts. Among the multitude of CpG sites investigated, a small subset has consistently emerged as highly informative across diverse populations, tissues, and platforms. This has prompted increased interest in developing simplified models that use only a few CpG sites to estimate biological age in a cost-effective, rapid, and scalable manner, with an acceptable loss of accuracy relative to more complex clocks.

Several simplified epigenetic clocks have been developed that rely on a limited number of CpG sites, typically between 3 and 8, measured using targeted, cost-effective techniques. For instance, a well-known example is Weidner’s clock, which estimates the age from the analysis of the methylation in three CpGs located in *ITGA2B*, *ASPA*, and *PDE4C*, quantified by bisulfite pyrosequencing, obtained a MAE < 5 years (Weidner et al. 2014). Similarly, a seven CpG model targeting sites in *ELOVL2*, *NHLRC1*, *AIM2*, *EDARADD*, *SIRT7*, and *TFAP2E*, measured by the EpiTYPER assay, reported a correlation of 0.89 with chronological age in the training data and 0.76 in test sets, with a mean absolute deviation (MAD) of approximately 4 and 5.8 years, respectively, in individuals aged 20–80 years (Gensous et al. 2022). More recently, Kim et al. developed EpiClock, an eight-CpG markers (ASPA, FHL2, MIR29B2CHG, Chr16q24.1, SLC12A5, SST, LDB2, and COL1A1) blood-based model optimized for high-throughput iPlex mass spectrometry. The model achieved an R^2^ of 0.93, a mean absolute deviation of 3.78 years, and very high inter-operator and intra-assay reproducibility, illustrating that reduced-CpG clocks can reach chronologic-age prediction errors in the 3—6 year range while remaining analytically robust (Kim et al. 2025) (Table 3).

**Table 3:** Comparison of minimalist epigenetic clocks using targeted techniques. Columns include clock name, publication year, correlation coefficient (R) between predicted and chronological age in validation cohorts, mean absolute error (MAE, years)chronological age range of validation subjects, number of training samples (N Training), number of validation samples (N Validation), number of CpG sites used in each model (N CpGs), number of genes (N Genes) and the targeted method used (Method)

Clock	Year	R	MAE	Age range	N training	N validation	N CpGs	N genes	Method
Weidner (Weidner et al. 2014)	2014	N/R^a^	4.5	N/R^a^	82	69	3	3	Bisulfite pyrosequencing
Gensous (Gensous et al. 2022)	2022	0.76	5.8^b^	20—80	~ 222^c^	~ 56^c^	7	6	EpiTYPER assay
EpiClock (Kim et al. 2025)	2025	0.97^d^	3.78	19—92	113	236	8	8	iPlex mass spectrometry
Ibáñez-Cabellos (Ibáñez-Cabellos et al. 2025)	2025	0.88	5.04	20—79	83	52	8	1	Bisulfite pyrosequencing

^a^ *N/R* Not reported in original publications

^b^ Reported as median absolute deviation (MAD) in the original publication (Gensous et al. 2022)

^c^ Derived from iterative 80/20 train–test splits of 278 individuals (Gensous et al. 2022)

^d^ Reported as R^2^ = 0.9392 in the original publication; r = √0.9392 ≈ 0.969 (Kim et al. 2025)

In this context, one gene, in particular, has gained increasing attention due to its strong and consistent correlation with chronological age and reproducible performance across predictive models: the ELOngation of Very Long chain fatty acids protein 2 (*ELOVL2*) (Zbieć-Piekarska et al. 2015; Garagnani et al. 2012; Hannum et al. 2013). It is important to note that other loci, such as *FHL2*, *KLF14*, and *TRIM59*, have also been repeatedly identified in reduced-CpG models, suggesting that a limited set of CpGs may capture key age-associated signals across independent studies (Paparazzo et al. 2025; Davydova et al. 2024; Gensous et al. 2022; Johnson et al. 2022). Functionally, *ELOVL2* encodes a fatty acid elongase involved in the biosynthesis of long-chain omega-3 and omega-6 polyunsaturated fatty acids, including docosahexaenoic acid (DHA), which are essential for membrane integrity, inflammation modulation, and neural function (Leonard et al. 2002; Fraser 2004). These biological processes have been associated with aging and age-related pathologies such as macular degeneration, supporting *ELOVL2*’s relevance as a biomarker in the context of age-related biological process.

The interest in *ELOVL2* as a relevant biomarker of aging began in 2012, when it was identified as one of the strongly age-correlated loci in a cohort of 501 individuals spanning 9 to 99 years (Garagnani et al. 2012). CpG sites within its promoter region, particularly cg16867657, exhibited a progressive hypermethylation pattern starting from early life, with a Spearman correlation of 0.92 with age (Garagnani et al. 2012). This association was subsequently validated in independent studies involving over 2,000 subjects, where *ELOVL2* emerged as the most age-predictive CpG site out of more than 137.000 analyzed, with correlation coefficients exceeding R = 0.95 (Zbieć-Piekarska et al. 2015).

Methylation levels at CpG islands within the promoter region of *ELOVL2* have proven to be among the most consistently associated epigenetic markers with age across diverse populations, tissues, and cell types (Zhu et al. 2018; Slieker et al. 2018). Studies have confirmed the correlation of *ELOVL2* methylation with age in blood, saliva, buccal samples, and other tissues, showing its potential as a cross-tissue marker for age estimation (Cruciani-Guglielmacci et al. 2017; Slieker et al. 2018). While genes such as *KLF14* and *TRIM59* also show epigenetic age associations (Jung et al. 2019), *ELOVL2* methylation is particularly robust, with some brain tissues like the cerebellum exhibiting lower methylation compared to skin, where its methylation increases with age (Slieker et al. 2018). Notably, the methylation status of *ELOVL2* can account for a substantial proportion of the predictive capacity in certain epigenetic clock models (Zbieć-Piekarska et al. 2015; Slieker et al. 2018).

Due to its consistency, *ELOVL2* has been incorporated into multiple generations of epigenetic clocks. This gene is one component of blood-based models, for example the Hannum clock (Hannum et al. 2013), and later it became a key element in streamlined forensic and clinical applications, including minimalist clocks requiring only 1 to 3 CpGs (Zbieć-Piekarska et al. 2015). Its methylation pattern has proven to be relatively stable across different tissues such as blood, fibroblasts, buccal epithelium, and even postmortem samples, making it one of the most versatile and reproducible markers available (Slieker et al. 2018). Some tissue-specific contexts, like those studied by McEwen, Lu, Hannum, and Shireby, have also confirmed its reliability (Shireby et al. 2020; McEwen et al. 2020; A. T. Lu et al. 2023; Hannum et al. 2013).

At the same time, recent work has highlighted that aggregating information across large numbers of CpG sites and applying principal-component-based transformations can markedly improve the test–retest reliability of multi-locus clocks. In these approaches, often referred to as principal component (PC)-based clocks, age estimates are derived from combinations of CpG signals rather than individual loci, thereby leveraging shared biological signal while reducing technical noise across thousands of sites.

Higgins-Chen et al. demonstrated that PC-bases implementations of widely used clocks reduce within-sample variability from differences of up to 9 years between technical replicates to less than 1 year, substantially enhancing their suitability for longitudinal and interventional studies (Higgins-Chen et al. 2022). More recently, a comprehensive benchmarking of 18 clocks by Sehgal et al. showed that PC-based clocks, particularly PCGrimAge and SystemsAge, maintained the highest technical reliability across different array positions and DNA extraction protocols, while only a subset of clocks achieved good biological reliability across repeated biological measurements (Sehgal et al. 2025).

Together, these fundings suggest that, when appropriately modeled, multi-CpG clocks can be highly robust to technical variability, and that reduced-CpG models do not inherently confer superior resistance to batch effects.

From a technical perspective, *ELOVL2* methylation-based models offer several practical advantages. They can be implemented using targeted assays such as pyrosequencing or MassARRAY, require fewer analytical targets and simpler bioinformatic workflows, and are therefore easier to deploy in laboratories without access to high-density methylation arrays or next-generation sequencing. Models based onELOVL2-related CpGs have achieved MAEs below 5.5 years, with high correlations reported in specific cohorts, indicating that they can approximate chronological age under controlled conditions (Zbieć-Piekarska et al. 2015).

Because these models rely on a small number of loci, they do not benefit from the same noise-averaging properties as multi-locus, pPC-based clocks, and their comparative robustness to batch effects, pre-analytical variation and cross-platform differences remains to be established in independent benchmarking studies (Higgins-Chen et al. 2022; Sehgal et al. 2025). In addition, most minimalist clocks to date have been evaluated primarily for chronological age prediction, often within relatively homogeneous cohorts, and less frequently in relation to health outcomes, mortality or responsiveness to interventions.

The evaluation of *ELOVL2* gene methylation can thus be used as a quantitative measure for age estimation, functioning as a minimalist clock that may help reduce costs and facilitate the use of DNA methylation technologies in large clinical trials or cohort studies (Crimmins et al. 2024; Fong et al. 2024). Age prediction using this approach can indicate whether an individual appears older or younger than expected for their chronological age, providing a simple index of age acceleration (Crimmins et al. 2024; Fong et al. 2024).

Several recent approaches have further explored ELOVL2-based clocks. For example, the EpiAgePublic model employs a small number of CpG sites within *ELOVL2* and was developed using large-scale DNA methylation datasets generated with array-based technologies (e.g., Illumina 450 K and EPIC), encompassing thousands of CpG sites. Although the final model focuses on three CpG sites within the *ELOVL2* gene, its training and validation were performed on publicly available datasets comprising 4,625 individuals across a wide age range. In this context, the integrated EpiAgePublic model achieved a correlation of approximately 0.87 in training and 0.93 in validation datasets with chronological age, outperforming individual CpG sites and approaching the performance of more complex multi-locus clocks. Importantly, these validation results were derived from array-based data and are therefore not directly comparable to targeted sequencing-based approaches (Cheishvili et al. 2025).

In contrast, bisulfite-converted DNA followed by targeted next-generation sequencing of three CpG sites within the *ELOVL2* gene was applied in independent cohorts, including Down syndrome and Alzheimer’s disease samples, to assess the applicability of the model in specific clinical contexts rather than to perform formal predictive validation. These analyses support the potential utility of EpiAgePublic in diverse research and clinical settings, including studies of environmental and pathological influences on biological aging (Cheishvili et al. 2025).

Another study described a minimalist, sex-specific epigenetic clock based on the methylation levels of CpG sites within the *ELOVL2* gene evaluated in buccal swab samples (Ibáñez-Cabellos et al. 2025). The model includes eight CpGs, with sex-stratified versions to account for potential biological differences in methylation patterns **(**Table 3**)**. In the evaluated cohorts, albeit of relatively limited size, the model showed correlations with chronological age of 0.88 for the non-sex-specific model and 0.85 and 0.89 for the female and male sex-specific models, respectively. These results are consistent with the performance reported for reduced-CpG clocks, with MAE of 5.04 years for the non-sex-specific model and 5.38 and 4.37 years for the female and male models, respectively (Table 3).

Current evidence that ELOVL2-only or very-low-CpG clocks capture multi-system “biological age” and predict age-related diseases, disability or mortality to the same extent as second-generation health- and mortality-trained clocks (such as PhenoAge, GrimAge or DunedinPACE) remains limited (Crimmins et al. 2024; Mavrommatis et al. 2025; Yamada 2025). Accordingly, these minimalist clocks are best viewed as complementary, cost-effective tools for chronologic age estimation and large-scale screening, rather than substitutes for more complex next-generation biomarkers when the primary goal is risk stratification or the evaluation of geroscience interventions (Crimmins et al. 2024; Belsky et al. 2022, 2020; Mavrommatis et al. 2025).

As with other reduced-CpG approaches, their applicability beyond chronological age estimation remains under investigation, particularly in relation to clinically relevant outcomes such as morbidity, mortality, or functional decline. Direct quantitative comparisons with commercial multi-locus clocks are further complicated by differences in tissue source, assay technology, age range and validation design.

Validation of theses simplified clocks is based on relatively small cohorts, often from a single center. Large external cohorts, cross-platform comparisons, and longitudinal studies will be required to establish their performance across populations, their biological reliability over time scales and their ability to predict clinically relevant outcomes (Higgins-Chen et al. 2022; Sehgal et al. 2025; Mavrommatis et al. 2025).

In this sense, ELOVL2-based minimalist clocks are best positioned as scalable tools for age estimation and screening in large cohorts, complementing rather than replacing more complex multi-locus biomarkers in clinical and geroscience research.

## Conclusion and future perspectives

With life expectancy rising globally, understanding and quantifying the biological underpinnings of aging has become a central challenge in biomedical research (Ferrucci et al. 2020). Among the various hallmarks of aging, epigenetic alterations, particularly DNA methylation changes, have emerged as one of the most robust and quantifiable biomarkers of biological age. Epigenetic clocks harness these methylation patterns to estimate biological aging and are increasingly used in population studies to predict disease risk, evaluate geroscience-guided interventions, and refine risk stratification strategies. While multiple generations of DNA methylation-based clocks have demonstrated strong associations with morbidity and mortality, their translation into routine clinical practice has been constrained by technical complexity, high assay costs, sensitivity to batch and platform effects, and the absence of consensus standards for clinical interpretation.

As research progresses, further refinement and rigorous validation of these tools will be essential to realize the potential of epigenetic biomarkers for promoting healthy aging and guiding personalized healthcare strategies across diverse populations. In parallel, the development of more accessible, standardized protocols for biological age estimation may facilitate broader use beyond specialized research centers. Accessible tools for estimating biological age could, in principle, empower individuals to monitor age-related trajectories and engage more proactively in health management, although recent position and consensus-style reviews caution that most clocks remain insufficiently reliable for individual-level clinical decision making (Ferrucci et al. 2020; Apsley et al. 2025).

Within this broader landscape of aging biomarkers, there is growing interest in minimalist, cost-conscious epigenetic clocks that aim to preserve useful predictive performance while simplifying laboratory and computational requirements. *ELOVL2* has repeatedly emerged as a key age-associated locus that is incorporated into many epigenetic clocks and, in some cases, supports stand-alone, reduced-CpG models that perform competitively against more complex predictors in large cohorts. Such reduced-CpG, targeted assays may mitigate some practical barriers associated with commercial array-based clocks, including reliance on high-density methylation arrays, complex bioinformatic pipelines, and the need for specialized computational infrastructure.

At the same time, minimalist clocks may be more vulnerable to technical noise and batch effects than high-dimensional or principal-component-based models, which deliberately average signal across many CpGs to bolster reliability (Higgins-Chen et al. 2022). Recent systematic evaluations have also shown that strong technical reproducibility does not necessarily guarantee high biological stability over short time scales, and that many existing clocks still display only moderate biological reliability in repeated measures across hours to days (Sehgal et al. 2025).

Consistent with these field-wide observations, current validations of *ELOVL2*-based clocks remain limited by relatively small sample sizes, single-center recruitment, and the lack of multi-platform and longitudinal assessments. A biological age estimator based solely on *ELOVL2* methylation should therefore be regarded as a promising, yet still exploratory, approach toward more practical and scalable tools for biological age assessment in settings where array-based clocks are not feasible, and its eventual integration into preventive medicine, clinical trials, and population studies will depend on further evidence of analytical and biological reliability, clinical validity, cost-effectiveness, and therefore should not be used to guide individual-level clinical decisions until these requirements are fulfilled (Apsley et al. 2025).

## Data Availability

No datasets were generated or analysed during the current study.

## References

[CR1] Rechsteiner, Cheyenne, Francesco Morandini, Kevin Perez, et al. (2022). ‘Development of a Novel Aging Clock Based on Chromatin Accessibility’. Preprint, Genomics, August 12. 10.1101/2022.08.11.502778.

[CR2] Apsley AT, Etzel L, Ye Q, Shalev I (2025) From population science to the clinic? Limits of epigenetic clocks as personal biomarkers. Epigenomics 17(18):1447–1461. 10.1080/17501911.2025.2603880 41403206 10.1080/17501911.2025.2603880 PMC12714307

[CR3] Argentieri MA, Xiao S, Bennett D et al (2024) Proteomic aging clock predicts mortality and risk of common age-related diseases in diverse populations. Nat Med 30(9):2450–2460. 10.1038/s41591-024-03164-7 39117878 10.1038/s41591-024-03164-7 PMC11405266

[CR4] Armstrong NJ, Mather KA, Thalamuthu A et al (2017) Aging, exceptional longevity and comparisons of the Hannum and Horvath epigenetic clocks. Epigenomics 9(5):689–700. 10.2217/epi-2016-0179 28470125 10.2217/epi-2016-0179

[CR5] Baker GT, Sprott RL (1988) Biomarkers of aging. Exp Gerontol 23(4–5):223–239. 10.1016/0531-5565(88)90025-3 3058488 10.1016/0531-5565(88)90025-3

[CR6] Bell CG, Lowe R, Adams PD et al (2019) DNA methylation aging clocks: challenges and recommendations. Genome Biol 20(1):249. 10.1186/s13059-019-1824-y 31767039 10.1186/s13059-019-1824-y PMC6876109

[CR7] Belsky DW, Moffitt TE, Cohen AA et al (2018) Eleven telomere, epigenetic clock, and biomarker-composite quantifications of biological aging: Do They measure the same thing? Am J Epidemiol 187(6):1220–1230. 10.1093/aje/kwx346 29149257 10.1093/aje/kwx346 PMC6248475

[CR8] Belsky DW, Caspi A, Arseneault L et al (2020) Quantification of the pace of biological aging in humans through a blood test, the DunedinPoAm DNA methylation algorithm. Elife 9:e54870. 10.7554/eLife.54870 32367804 10.7554/eLife.54870 PMC7282814

[CR9] Belsky DW, Caspi A, Corcoran DL et al (2022) DunedinPACE, a DNA methylation biomarker of the pace of aging. Elife 11:e73420. 10.7554/eLife.73420 35029144 10.7554/eLife.73420 PMC8853656

[CR10] Belsky, Daniel W, Avshalom Caspi, Renate Houts, et al. 2015. Quantification of biological aging in young adults. Proceedings of the national academy of sciences 112 (30) 10.1073/pnas.1506264112 PMC4522793 26150497

[CR11] Benayoun BéréniceA, Pollina EA, Brunet A (2015) Epigenetic regulation of ageing: linking environmental inputs to genomic stability. Nat Rev Mol Cell Biol 16(10):593–610. 10.1038/nrm4048 26373265 10.1038/nrm4048 PMC4736728

[CR12] Benayoun BéréniceA, Pollina EA, Singh PP et al (2019) Remodeling of epigenome and transcriptome landscapes with aging in mice reveals widespread induction of inflammatory responses. Genome Res 29(4):697–709. 10.1101/gr.240093.118 30858345 10.1101/gr.240093.118 PMC6442391

[CR13] Benjamin H (1947) Biologic versus chronologic age. J Gerontol 2(3):217–227. 10.1093/geronj/2.3.217 20264999 10.1093/geronj/2.3.217

[CR14] Beynon RA, Ingle SM, Langdon R et al (2022) Epigenetic biomarkers of ageing are predictive of mortality risk in a longitudinal clinical cohort of individuals diagnosed with oropharyngeal cancer. Clin Epigenetics 14(1):1. 10.1186/s13148-021-01220-4 34980250 10.1186/s13148-021-01220-4 PMC8725548

[CR15] Sehgal, Raghav, Daniel Borrus, John Gonzalez, Yaroslav Markov, ADNI, and Albert Higgins-Chen. (2025). Biological versus technical reliability of epigenetic clocks and implications for disease prognosis and intervention response. *bioRxiv [Preprint]*, October 14. 10.1101/2025.10.13.682176. 10.1111/acel.70635 PMC13418614 42525215

[CR16] Bozack AK, Khodasevich D, Nwanaji-Enwerem JC et al (2025) One-carbon metabolism-related compounds are associated with epigenetic aging biomarkers: results from the cross-sectional national health and nutrition examination survey 1999–2002. Am J Clin Nutr 122(2):413–423. 10.1016/j.ajcnut.2025.05.029 40456316 10.1016/j.ajcnut.2025.05.029 PMC12405778

[CR17] Cheishvili D, Do Carmo S, Caraci F, Grasso M, Cuello AC, Szyf M (2025) EpiAge: a next-generation sequencing-based ELOVL2 epigenetic clock for biological age assessment in saliva and blood across health and disease. Aging 17(1):131–160. 10.18632/aging.206188 39853302 10.18632/aging.206188 PMC11810066

[CR18] Chen L, Tan K-L, Xu J et al (2024) Exploring multi-omics and clinical characteristics linked to accelerated biological aging in Asian women of reproductive age: insights from the S-PRESTO Study. Genome Med 16(1):128. 10.1186/s13073-024-01403-7 39516835 10.1186/s13073-024-01403-7 PMC11549770

[CR19] Ciccarone F, Tagliatesta S, Caiafa P, Zampieri M (2018) DNA methylation dynamics in aging: How far are we from understanding the mechanisms? Mech Ageing Dev 174(September):3–17. 10.1016/j.mad.2017.12.002 29268958 10.1016/j.mad.2017.12.002

[CR20] Crimmins EM, Thyagarajan B, Levine ME, Weir DR, Faul J (2021) Associations of age, sex, race/ethnicity, and education with 13 epigenetic clocks in a nationally representative U.S. sample: the health and retirement study. J Gerontol A Biol Sci Med Sci 76(6):1117–1123. 10.1093/gerona/glab016 33453106 10.1093/gerona/glab016 PMC8140049

[CR21] Crimmins EM, Klopack ET, Kim JK (2024) Generations of epigenetic clocks and their links to socioeconomic status in the health and retirement study. Epigenomics 16(14):1031–1042. 10.1080/17501911.2024.2373682 39023350 10.1080/17501911.2024.2373682 PMC11404624

[CR22] Cruciani-Guglielmacci C, Bellini L, Denom J et al (2017) Molecular phenotyping of multiple mouse strains under metabolic challenge uncovers a role for Elovl2 in glucose-induced insulin secretion. Mol Metab 6(4):340–351. 10.1016/j.molmet.2017.01.009 28377873 10.1016/j.molmet.2017.01.009 PMC5369210

[CR23] Davydova E, Perenkov A, Vedunova M (2024) Building minimized epigenetic clock by iPlex MassARRAY Platform. Genes Basel 15(4):425. 10.3390/genes15040425 38674360 10.3390/genes15040425 PMC11049545

[CR24] Dhingra R, Nwanaji-Enwerem JC, Samet M, Ward-Caviness CK (2018) DNA methylation age—environmental influences, health impacts, and its role in environmental epidemiology. Curr Environ Health Rep 5(3):317–327. 10.1007/s40572-018-0203-2 30047075 10.1007/s40572-018-0203-2 PMC6173330

[CR25] Di Lena P, Nardini C, Pellegrini M (2022) Editorial: computational methods for analysis of DNA methylation data. Front Bioinform 2(June):926066. 10.3389/fbinf.2022.926066 36304337 10.3389/fbinf.2022.926066 PMC9580921

[CR26] Duan R, Fu Q, Sun Y, Li Q (2022) Epigenetic clock: A promising biomarker and practical tool in aging. Ageing Res Rev 81(November):101743. 10.1016/j.arr.2022.101743 36206857 10.1016/j.arr.2022.101743

[CR27] Durso DF, Bacalini MG, Sala C et al (2017) Acceleration of leukocytes’ epigenetic age as an early tumor and sex-specific marker of breast and colorectal cancer. Oncotarget 8(14):23237–23245. 10.18632/oncotarget.15573 28423572 10.18632/oncotarget.15573 PMC5410300

[CR28] Ferrucci L, Gonzalez-Freire M, Fabbri E et al (2020) Measuring biological aging in humans: a quest. Aging Cell 19(2):e13080. 10.1111/acel.13080 31833194 10.1111/acel.13080 PMC6996955

[CR29] Field AE, Robertson NA, Wang T, Havas A, Ideker T, Adams PD (2018) DNA methylation clocks in aging: categories, causes, and consequences. Mol Cell 71(6):882–895. 10.1016/j.molcel.2018.08.008 30241605 10.1016/j.molcel.2018.08.008 PMC6520108

[CR30] Fong S, Pabis K, Latumalea D et al (2024) Principal component-based clinical aging clocks identify signatures of healthy aging and targets for clinical intervention. Nature Aging 4(8):1137–1152. 10.1038/s43587-024-00646-8 38898237 10.1038/s43587-024-00646-8 PMC11333290

[CR31] Fraga MF, Esteller M (2007) Epigenetics and aging: The targets and the marks. Trends Genet 23(8):413–418. 10.1016/j.tig.2007.05.008 17559965 10.1016/j.tig.2007.05.008

[CR32] Fraga MF, Ballestar E, Paz MF et al (2005) Epigenetic differences arise during the lifetime of monozygotic twins. Proc Natl Acad Sci 102(30):10604–10609. 10.1073/pnas.0500398102 16009939 10.1073/pnas.0500398102 PMC1174919

[CR33] Fraser P (2004) The biosynthesis and nutritional uses of carotenoids. Prog Lipid Res 43(3):228–265. 10.1016/j.plipres.2003.10.002 15003396 10.1016/j.plipres.2003.10.002

[CR34] Frenck RW, Blackburn EH, Shannon KM (1998) The rate of telomere sequence loss in human leukocytes varies with age. Proc Natl Acad Sci 95(10):5607–5610. 10.1073/pnas.95.10.5607 9576930 10.1073/pnas.95.10.5607 PMC20425

[CR35] Galkin F, Mamoshina P, Kochetov K, Sidorenko D, Zhavoronkov A (2021) DeepMAge: a methylation aging clock developed with deep learning. Aging Dis 12(5):1252. 10.14336/AD.2020.1202 34341706 10.14336/AD.2020.1202 PMC8279523

[CR36] Garagnani P, Bacalini MG, Pirazzini C et al (2012) Methylation of *ELOVL 2* gene as a new epigenetic marker of age. Aging Cell 11(6):1132–1134. 10.1111/acel.12005 23061750 10.1111/acel.12005

[CR37] Gensous N, Sala C, Pirazzini C et al (2022) A targeted epigenetic clock for the prediction of biological age. Cells 11(24):4044. 10.3390/cells11244044 36552808 10.3390/cells11244044 PMC9777448

[CR38] Grabowska W, Sikora E, Bielak-Zmijewska A (2017) Sirtuins, a promising target in slowing down the ageing process. Biogerontology 18(4):447–476. 10.1007/s10522-017-9685-9 28258519 10.1007/s10522-017-9685-9 PMC5514220

[CR39] Hannum G, Guinney J, Zhao L et al (2013) Genome-wide methylation profiles reveal quantitative views of human aging rates. Mol Cell 49(2):359–367. 10.1016/j.molcel.2012.10.016 23177740 10.1016/j.molcel.2012.10.016 PMC3780611

[CR40] Higgins-Chen AT, Thrush KL, Wang Y et al (2022) A computational solution for bolstering reliability of epigenetic clocks: implications for clinical trials and longitudinal tracking. Nat Aging 2(7):644–661. 10.1038/s43587-022-00248-2 36277076 10.1038/s43587-022-00248-2 PMC9586209

[CR41] Horvath S (2013) DNA methylation age of human tissues and cell types. Genome Biol 14(10):3156. 10.1186/gb-2013-14-10-r115 10.1186/gb-2013-14-10-r115 PMC4015143 24138928

[CR42] Horvath S, Raj K (2018) DNA methylation-based biomarkers and the epigenetic clock theory of ageing. Nat Rev Genet 19(6):371–384. 10.1038/s41576-018-0004-3 29643443 10.1038/s41576-018-0004-3

[CR43] Huan T, Chen G, Liu C et al (2018) Age‐associated micro RNA expression in human peripheral blood is associated with all‐cause mortality and age‐related traits. Aging Cell 17(1):e12687. 10.1111/acel.12687 29044988 10.1111/acel.12687 PMC5770777

[CR44] Ibáñez-Cabellos JS, Sandoval J, Pallardó FV, García-Giménez JL, Mena-Molla S (2025) A sex-specific minimal CpG-based model for biological aging using ELOVL2 methylation analysis. Int J Mol Sci 26(7):3392. 10.3390/ijms26073392 40244262 10.3390/ijms26073392 PMC11989821

[CR45] Ikram MA (2024) The use and misuse of “biological aging” in health research. Nat Med 30(11):3045–3045. 10.1038/s41591-024-03297-9 39375458 10.1038/s41591-024-03297-9

[CR46] Janssens GE, Van Dongen J, Ligthart L, De Geus EJC, Salomons GS (2025) Nutritional associations with decelerated epigenetic aging: vegan diet in a Dutch population. Clin Epigenetics 17(1):133. 10.1186/s13148-025-01934-9 40731356 10.1186/s13148-025-01934-9 PMC12305949

[CR47] Johnson AA, Torosin NS, Shokhirev MN, Cuellar TL (2022) A set of common buccal CpGs that predict epigenetic age and associate with lifespan-regulating genes. iScience 25(11):105304. 10.1016/j.isci.2022.105304 36304118 10.1016/j.isci.2022.105304 PMC9593711

[CR48] Jung S-E, Lim SM, Hong SR, Lee EH, Shin K-J, Lee HY (2019) DNA methylation of the ELOVL2, FHL2, KLF14, C1orf132/MIR29B2C, and TRIM59 genes for age prediction from blood, saliva, and buccal swab samples. Forensic Sci Int Genet 38:1–8. 10.1016/j.fsigen.2018.09.010 30300865 10.1016/j.fsigen.2018.09.010

[CR49] Jylhävä J, Pedersen NL, Hägg S (2017) Biological age predictors. EBioMedicine 21:29–36. 10.1016/j.ebiom.2017.03.046 28396265 10.1016/j.ebiom.2017.03.046 PMC5514388

[CR50] Kerepesi C, Gladyshev VN (2023) Intersection clock reveals a rejuvenation event during human embryogenesis. Aging Cell 22(10):e13922. 10.1111/acel.13922 37786333 10.1111/acel.13922 PMC10577537

[CR51] Kim H, Park A-H, Kwon M, Lee KJ, Kim M-J, Lee M-S (2025) EpiClock; biological age measurement from blood DNA methylation using a minimal CpG marker set for high-throughput iPlex mass spectrometry assay for screening in drug development and population health. Exp Gerontol 211:112918. 10.1016/j.exger.2025.112918 41067661 10.1016/j.exger.2025.112918

[CR52] Kittana, Monia, Vasso Apostolopoulos, and Lily Stojanovska. (2024). ‘The role of calorie restriction in modifying the ageing process through the regulation of SIRT1 expression’. In *Biochemistry and Cell Biology of Ageing: Part V, Anti-Ageing Interventions*, edited by Viktor I. Korolchuk and J. Robin Harris, vol. 107. Subcellular Biochemistry. Springer Nature Switzerland 10.1007/978-3-031-66768-8_8 39693024

[CR53] Krištić J, Vučković F, Menni C et al (2014) Glycans are a novel biomarker of chronological and biological ages. J Gerontol: Ser A 69(7):779–789. 10.1093/gerona/glt190 10.1093/gerona/glt190 PMC4049143 24325898

[CR54] Lai T-P, Wright WE, Shay JW (2018) Comparison of telomere length measurement methods. Philos Trans R Soc Lond B Biol Sci 373(1741):20160451. 10.1098/rstb.2016.0451 29335378 10.1098/rstb.2016.0451 PMC5784071

[CR55] Leonard AE, Kelder B, Bobik EG et al (2002) Identification and expression of mammalian long‐chain PUFA elongation enzymes. Lipids 37(8):733–740. 10.1007/s11745-002-0955-6 12371743 10.1007/s11745-002-0955-6

[CR56] Levine ME (2020) Assessment of epigenetic clocks as biomarkers of aging in basic and population research. J Gerontol: Ser A 75(3):463–465. 10.1093/gerona/glaa021 10.1093/gerona/glaa021 PMC7328198 31995162

[CR57] Levine ME, Lu AT, Quach A et al (2018) An epigenetic biomarker of aging for lifespan and healthspan. Aging 10(4):573–591. 10.18632/aging.101414 29676998 10.18632/aging.101414 PMC5940111

[CR58] Levy MZ, Allsopp RC, Futcher AB, Greider CW, Harley CB (1992) Telomere end-replication problem and cell aging. J Mol Biol 225(4):951–960. 10.1016/0022-2836(92)90096-3 1613801 10.1016/0022-2836(92)90096-3

[CR59] Li Z, Zhang W, Duan Y et al (2023) Progress in biological age research. Front Public Health 11:1074274. 10.3389/fpubh.2023.1074274 37124811 10.3389/fpubh.2023.1074274 PMC10130645

[CR60] Longo VD, Kennedy BK (2006) Sirtuins in aging and age-related disease. Cell 126(2):257–268. 10.1016/j.cell.2006.07.002 16873059 10.1016/j.cell.2006.07.002

[CR61] López-Otín C, Blasco MA, Partridge L, Serrano M, Kroemer G (2013) The hallmarks of aging. Cell 153(6):1194–1217. 10.1016/j.cell.2013.05.039 23746838 10.1016/j.cell.2013.05.039 PMC3836174

[CR62] López-Otín C, Blasco MA, Partridge L, Serrano M, Kroemer G (2023) Hallmarks of aging: an expanding universe. Cell 186(2):243–278. 10.1016/j.cell.2022.11.001 36599349 10.1016/j.cell.2022.11.001

[CR63] Lu AT, Quach A, Wilson JG et al (2019) DNA methylation GrimAge strongly predicts lifespan and healthspan. Aging 11(2):303–327. 10.18632/aging.101684 30669119 10.18632/aging.101684 PMC6366976

[CR64] Lu AT, Fei Z, Haghani A et al (2023) Universal DNA methylation age across mammalian tissues. Nat Aging 3(9):1144–1166. 10.1038/s43587-023-00462-6 37563227 10.1038/s43587-023-00462-6 PMC10501909

[CR65] Macdonald-Dunlop E, Taba N, Klarić L et al (2022) A catalogue of Omics biological ageing clocks reveals substantial commonality and associations with disease risk. Aging 14(2):623–659. 10.18632/aging.203847 35073279 10.18632/aging.203847 PMC8833109

[CR66] Mak JKL, Karlsson IK, Tang B et al (2024) Temporal dynamics of epigenetic aging and frailty from midlife to old age. J Gerontol: Ser A 79(10):glad251. 10.1093/gerona/glad251 10.1093/gerona/glad251 PMC11421301 37889476

[CR67] Mathur A, Taurin S, Alshammary S (2024) New insights into methods to measure biological age: a literature review. Front Aging 5:1395649. 10.3389/fragi.2024.1395649 39743988 10.3389/fragi.2024.1395649 PMC11688636

[CR68] Mavrommatis C, Belsky DW, Ying K et al (2025) An unbiased comparison of 14 epigenetic clocks in relation to 174 incident disease outcomes. Nat Commun 16(1):11164. 10.1038/s41467-025-66106-y 41402269 10.1038/s41467-025-66106-y PMC12708718

[CR69] McCrory C, Fiorito G, Hernandez B et al (2021) GrimAge outperforms other epigenetic clocks in the prediction of age-related clinical phenotypes and all-cause mortality. J Gerontol A Biol Sci Med Sci 76(5):741–749. 10.1093/gerona/glaa286 33211845 10.1093/gerona/glaa286 PMC8087266

[CR70] McEwen LM, O’Donnell KJ, McGill MG et al (2020) The PedBE clock accurately estimates DNA methylation age in pediatric buccal cells. Proc Natl Acad Sci U S A 117(38):23329–23335. 10.1073/pnas.1820843116 31611402 10.1073/pnas.1820843116 PMC7519312

[CR71] McIntyre RL, Rahman M, Vanapalli SA, Houtkooper RH, Janssens GE (2021) Biological age prediction from wearable device movement data identifies nutritional and pharmacological interventions for healthy aging. Front Aging 2:708680. 10.3389/fragi.2021.708680 35822021 10.3389/fragi.2021.708680 PMC9261299

[CR72] Miao K, Hong X, Cao W et al (2024) Association between epigenetic age and type 2 diabetes mellitus or glycemic traits: a longitudinal twin study. Aging Cell 23(7):e14175. 10.1111/acel.14175 38660768 10.1111/acel.14175 PMC11258448

[CR73] Niederberger C, Vermeersch A, Davidhi F et al (2023) Wearable sweat analysis to determine biological age. Trends Biotechnol 41(9):1113–1116. 10.1016/j.tibtech.2023.02.001 36822913 10.1016/j.tibtech.2023.02.001

[CR74] Noroozi R, Ghafouri-Fard S, Pisarek A et al (2021) DNA methylation-based age clocks: from age prediction to age reversion. Ageing Res Rev 68:101314. 10.1016/j.arr.2021.101314 33684551 10.1016/j.arr.2021.101314

[CR75] Oh H-H, Rutledge J, Nachun D et al (2023) Organ aging signatures in the plasma proteome track health and disease. Nature 624(7990):164–172. 10.1038/s41586-023-06802-1 38057571 10.1038/s41586-023-06802-1 PMC10700136

[CR76] Paparazzo E, Aceto MA, Cassano TS et al (2025) Reproducibility and validation of a targeted and flexible epigenetic clock for forensic applications. Forensic Sci Int 369(April):112409. 10.1016/j.forsciint.2025.112409 39983295 10.1016/j.forsciint.2025.112409

[CR77] Pyrkov TV, Sokolov IS, Fedichev PO (2021) Deep longitudinal phenotyping of wearable sensor data reveals independent markers of longevity, stress, and resilience. Aging 13(6):7900–7913. 10.18632/aging.202816 33735108 10.18632/aging.202816 PMC8034931

[CR78] Roig-Genoves JV, García-Giménez JL, Mena-Molla S (2024) A miRNA-based epigenetic molecular clock for biological skin-age prediction. Arch Dermatol Res 316(6):326. 10.1007/s00403-024-03129-3 38822910 10.1007/s00403-024-03129-3 PMC11144124

[CR79] Ryan JM, Cristofalo VJ (1972) Histone acetylation during aging of human cells in culture. Biochem Biophys Res Commun 48(4):735–742. 10.1016/0006-291X(72)90668-7 4636644 10.1016/0006-291x(72)90668-7

[CR80] Shireby GL, Davies JP, Francis PT et al (2020) Recalibrating the epigenetic clock: Implications for assessing biological age in the human cortex. Brain 143(12):3763–3775. 10.1093/brain/awaa334 33300551 10.1093/brain/awaa334 PMC7805794

[CR81] Shokhirev MN, Torosin NS, Kramer DJ, Johnson AA, Cuellar TL (2024) CheekAge: A next-generation buccal epigenetic aging clock associated with lifestyle and health. GeroScience 46(3):3429–3443. 10.1007/s11357-024-01094-3 38441802 10.1007/s11357-024-01094-3 PMC11009193

[CR82] Slieker RC, Relton CL, Gaunt TR, Slagboom PE, Heijmans BT (2018) Age-related DNA methylation changes are tissue-specific with ELOVL2 promoter methylation as exception. Epigenetics Chromatin 11(1):25. 10.1186/s13072-018-0191-3 29848354 10.1186/s13072-018-0191-3 PMC5975493

[CR83] Sugden K, Caspi A, Elliott ML et al (2022) Association of pace of aging measured by blood-based DNA methylation with age-related cognitive impairment and dementia. Neurology 99(13):e1402–e1413. 10.1212/WNL.0000000000200898 35794023 10.1212/WNL.0000000000200898 PMC9576288

[CR84] Tay J, Wang W, Guan L et al (2025) The association of physical function and physical performance with DNA methylation clocks in oldest-old living in Singapore—the SG90 cohort. J Gerontol A Biol Sci Med Sci 80(4):glaf022. 10.1093/gerona/glaf022 39869450 10.1093/gerona/glaf022

[CR85] Vaiserman A, Krasnienkov D (2021) Telomere length as a marker of biological age: state-of-the-art, open issues, and future perspectives. Front Genet. 10.3389/fgene.2020.630186 33552142 10.3389/fgene.2020.630186 PMC7859450

[CR86] Wang Y, Yuan Q, Xie L (2018) Histone modifications in aging: the underlying mechanisms and implications. Curr Stem Cell Res Ther 13(2):125–135. 10.2174/1574888X12666170817141921 28820059 10.2174/1574888X12666170817141921

[CR87] Weidner CI, Lin Q, Koch CM et al (2014) Aging of blood can be tracked by DNA methylation changes at just three CpG sites. Genome Biol 15(2):R24. 10.1186/gb-2014-15-2-r24 24490752 10.1186/gb-2014-15-2-r24 PMC4053864

[CR88] Whitman ET, Ryan CP, Abraham WC et al (2024) A blood biomarker of the pace of aging is associated with brain structure: Replication across three cohorts. Neurobiol Aging 136(April):23–33. 10.1016/j.neurobiolaging.2024.01.008 38301452 10.1016/j.neurobiolaging.2024.01.008 PMC11017787

[CR89] Wu Q, Bosanac M, Shokhirev MN, Raffington L (2025) Probing buccal-customized epigenetic clocks to investigate socioeconomic and health disparities. GeroScience. 10.1007/s11357-025-01907-z 41073833 10.1007/s11357-025-01907-z PMC13355981

[CR90] Yamada H (2025) Epigenetic clocks and EpiScore for preventive medicine: Risk stratification and intervention models for age-related diseases. J Clin Med 14(10):3604. 10.3390/jcm14103604 40429598 10.3390/jcm14103604 PMC12112696

[CR91] Yi S-J, Kim K (2020) New insights into the role of histone changes in aging. Int J Mol Sci 21(21):8241. 10.3390/ijms21218241 33153221 10.3390/ijms21218241 PMC7662996

[CR92] Yu HJ, Byun YH, Park C-K (2024) Techniques for assessing telomere length: a methodological review. Comput Struct Biotechnol J 23:1489–1498. 10.1016/j.csbj.2024.04.011 38633384 10.1016/j.csbj.2024.04.011 PMC11021795

[CR93] Zbieć-Piekarska R, Spólnicka M, Kupiec T et al (2015) Development of a forensically useful age prediction method based on DNA methylation analysis. Forensic Sci Int Genet 17:173–179. 10.1016/j.fsigen.2015.05.001 26026729 10.1016/j.fsigen.2015.05.001

[CR94] Zhang S, Wang Z, Wang Y et al (2024) A metabolomic profile of biological aging in 250,341 individuals from the UK Biobank. Nat Commun 15(1):8081. 10.1038/s41467-024-52310-9 39278973 10.1038/s41467-024-52310-9 PMC11402978

[CR95] Zheng Z, Li J, Liu T et al (2024) DNA methylation clocks for estimating biological age in Chinese cohorts. Protein Cell 15(8):575–593. 10.1093/procel/pwae011 38482631 10.1093/procel/pwae011 PMC11259550

[CR96] Zhu T, Zheng SC, Paul DS, Horvath S, Teschendorff AE (2018) Cell and tissue type independent age-associated DNA methylation changes are not rare but common. Aging Albany NY 10(11):3541–3557. 10.18632/aging.101666 30482885 10.18632/aging.101666 PMC6286821

